# Supplementary material for: Reprogramming Human Adult Fibroblasts into GABAergic Interneurons
Source: Cells. 2021 Dec 8;10(12):3450. doi: 10.3390/cells10123450 (PMC8699824; doi:10.3390/cells10123450)
Supplement: Supplementary file 1 [file cells-10-03450-s001.zip › cells-1457952-supplementary.pdf]

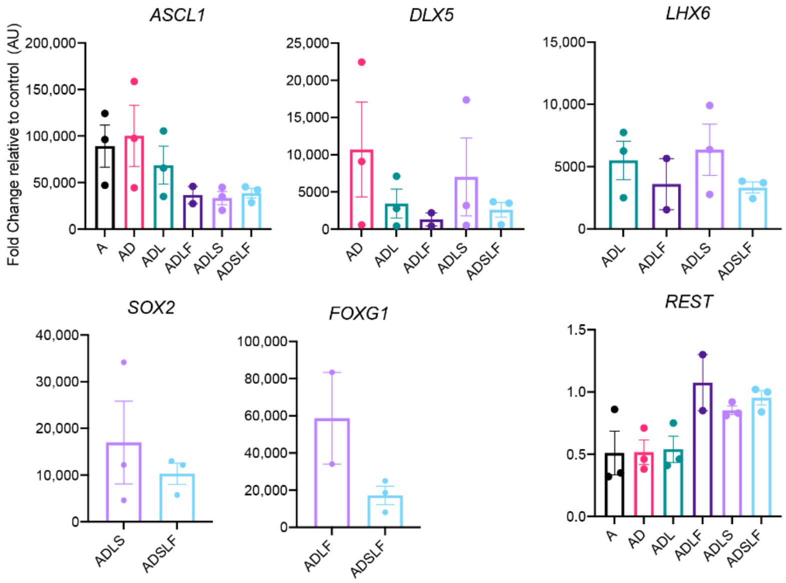

Figure S1. RT-qPCR upregulation of transcription factors and downregulation of REST compared to fibroblast control across all conditions at day 25 (n=2-3).

Table S1. List of primers used in this study.

Gene	Gene Name	Primer sequence 5'->3' (Fwd/Rev)
ACTB	Beta-actin	CCTTGCACATGCCGGAG<br>GCACAGAGCCTCGCCTT
ARX	Aristaless Related Homeobox	CCTGAGCACTTTCCTCGGAGCG<br>TGGAAAAGAGCCTGCCGAATGCC
ASCL1	Achaete-Scute Family BHLH Transcription Factor 1	WPRE-CATCTCCCCCAACTACTCCA<br>WPRE-TCTGGGCTAAGAGGGTCGTA
CALB1	Calbindin 1	TGGCTCCATTTCGACGCTGACG<br>ATCCAGCCTTCTTTCGCGCCTG
CALB2 (CALRETININ)	Calbindin 2	TGGAGGCTTGGCGGAAGTACGA<br>CCGGTTCGCCTTCTTCAGCAGG
DLX5	Distal-Less Homeobox 5	WPRE-GCCTCCGGGACACTCTATTA<br>WPRE-CAGTTTTCCGAACTTCCCCAT
PPP1R1B (DARPP32)	Protein Phosphatase 1 Regulatory Inhibitor Subunit 1B	GAGAGCCTCAGGAGAGGGGCAC<br>AGGTGGTGTGTAGGCACAGGGG
FOXG1	Forkhead Box G1	WPRE-AGGGGTCTTCTTCCAACCCT<br>WPRE-GCAGCGTATCCACATAGCGT
FSP1	Fibroblast-specific protein 1	GATGAGCAACTTGGACAGCAA<br>CTGGGCTGCTTATCTGGGAAG
GAD2 (GAD65)	Glutamate decarboxylase 2	ATCCTCACGACTCAGCTCCC<br>GAGCTTTAAAAGAGACCGGGACT
GAPDH	Glyceraldehyde-3-phosphate dehydrogenase	TTGAGGTCAATGAAGGGGTC<br>GAAGGTGAAGGTCGGAGTCA
LHX6	LIM Homeobox 6	WPRE- CACTTCCGCATCTGCCCGT<br>WPRE-CGCAGCTTGGACACTGGATCT
NKX2.1	NK2 homeobox 1	AGGGCGGGGCACAGATTGGA<br>GCTGGCAGAGTGTGCCCAGA
NPY	Neuropeptide Y	TGTTCCCAGAACTCGGCTTG<br>TGCATTGGTAGGATGGGTGG
MAP2	Microtubule Associated Protein 2	CCGTGTGGACCATGGGGCTG<br>GTCGTCGGGGTGATGCCACG

PVALB	Parvalbumin	TGCAGGATGTCGATGACAGA<br>TTTCTTCAGGCCGACCATTT
REST	RE1-Silencing Transcription factor	AAATGTGGCCTTAACTGGGGAA<br>TCTGTCTTTCTTCACCGACCAG
SOX2	SRY-box transcription factor 2	WPRE-TCGCACATGTGAGGATCCAA<br>WPRE-AGCGTAAAAGGAGCAACATAGT
SST	Somatostatin	CAAGCCGCTTTAGGAGCGAG<br>AGGCGGCAGGACAGCATCT
SYN1	Synapsin 1	CCCGTGGTTGTGAAGA TGGGGC<br>TGCCACGACACTTGCGATGTCC
TBR1	T-Box Brain Transcription Factor 1	TCGTCCCCGCTCAAGAGCGA<br>CCTTGGCGCAGTTCTTCTCGCA
TUBB3	Tubulin Beta 3 Class III	GGCCTTTGGACATCTCTTCA<br>ATACTCCTCACGCACCTTGC
SLC17A7 (VGLUT1)	Solute Carrier Family 17 Member 7	AATAACAGCACGACCCACCGCG<br>AGCCGTGTATGAGGCCGACAGT
VIP	Vasoactive intestinal peptide	TCTCACAGACTTCGGCATGG<br>TCATTTGCTCCCTCAAAGGGT

Table S2. List of primary antibodies used in this study.

Marker	Specificity	Source (cat. #)	Dilution
**TUJ1**	Mouse	Biolegend (801201)	1:1000
**MAP2**	Chicken	Abcam (ab5392)	1:2000
**CALB1**	Rabbit	Swant (CB38)	1:500
**CALB2**	Rabbit	Abcam (ab702)	1:200
**GABA**	Rabbit	Sigma (A2052)	1:2000
**PV**	Rabbit	Swant (PV27)	1:500
**PV**	Mouse	Sigma (P3088)	1:2000
